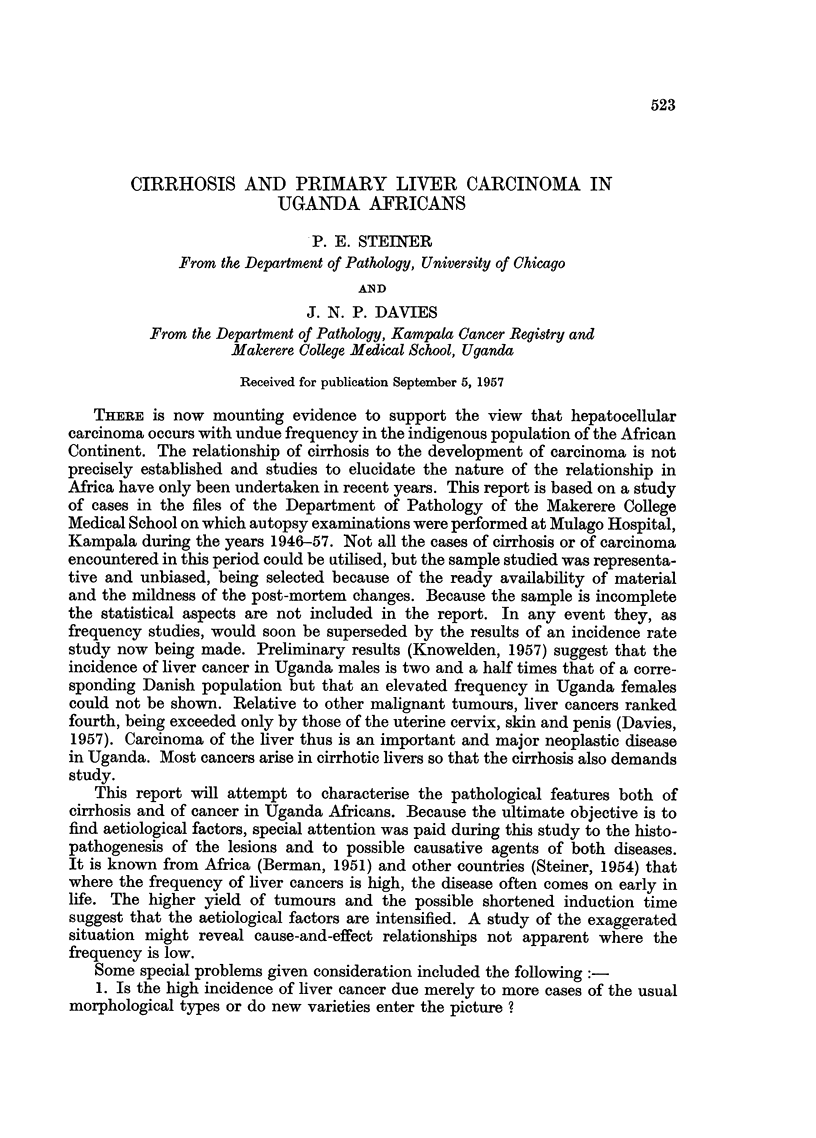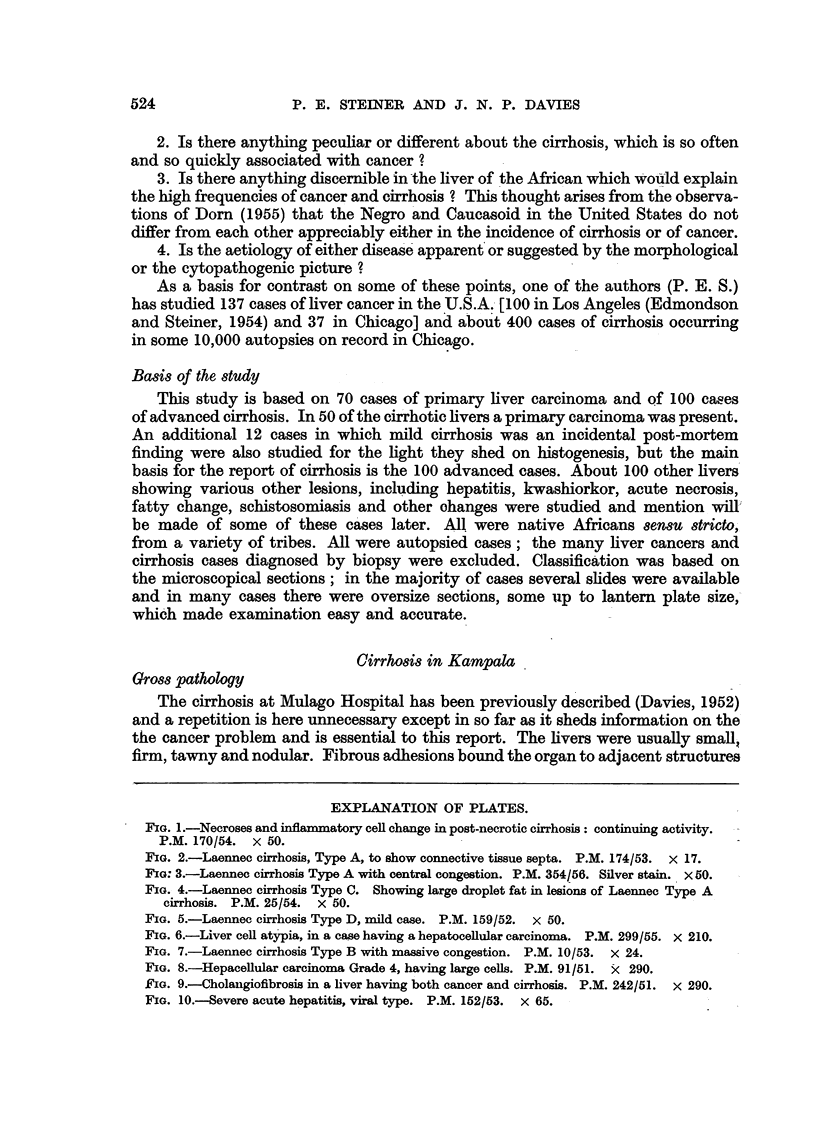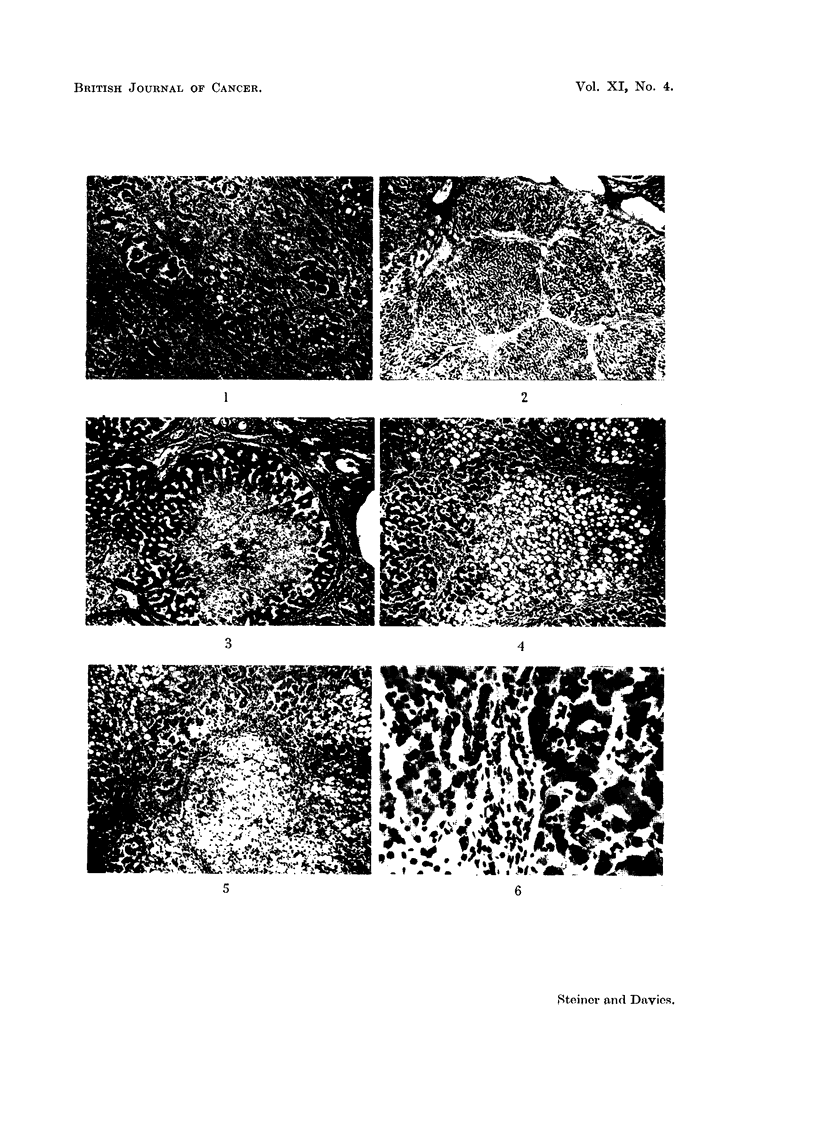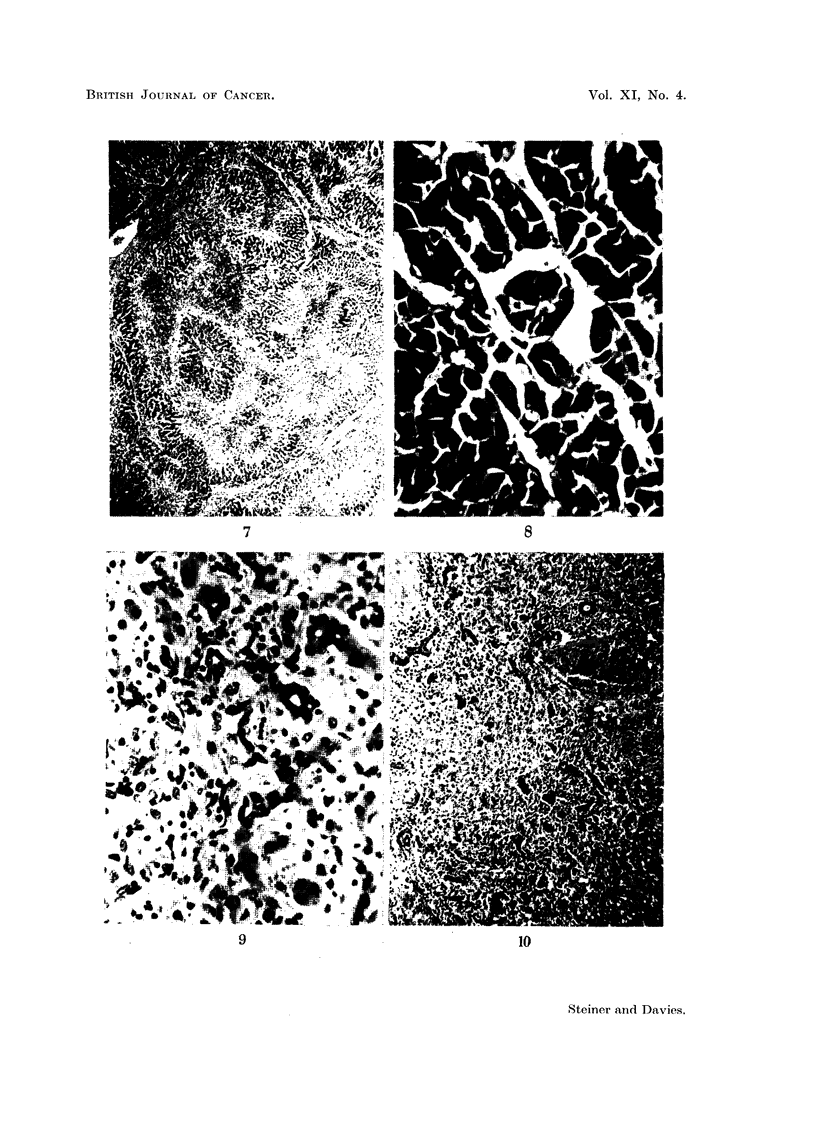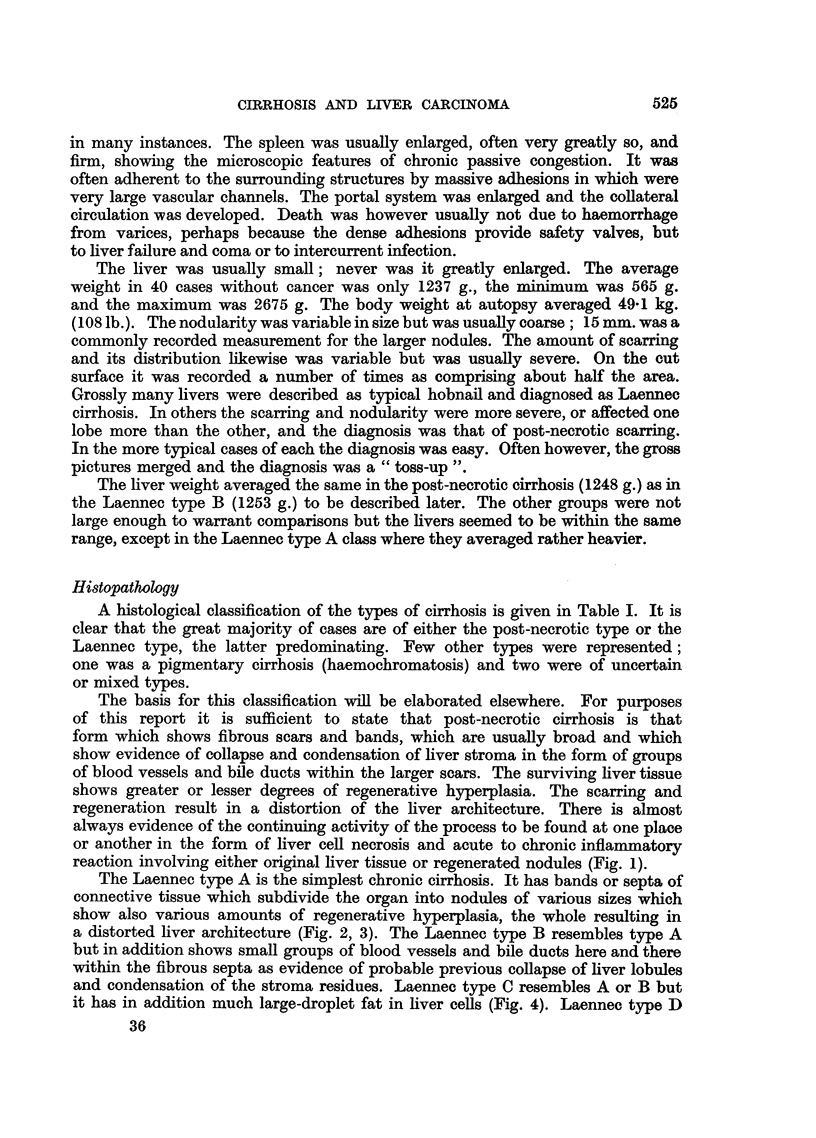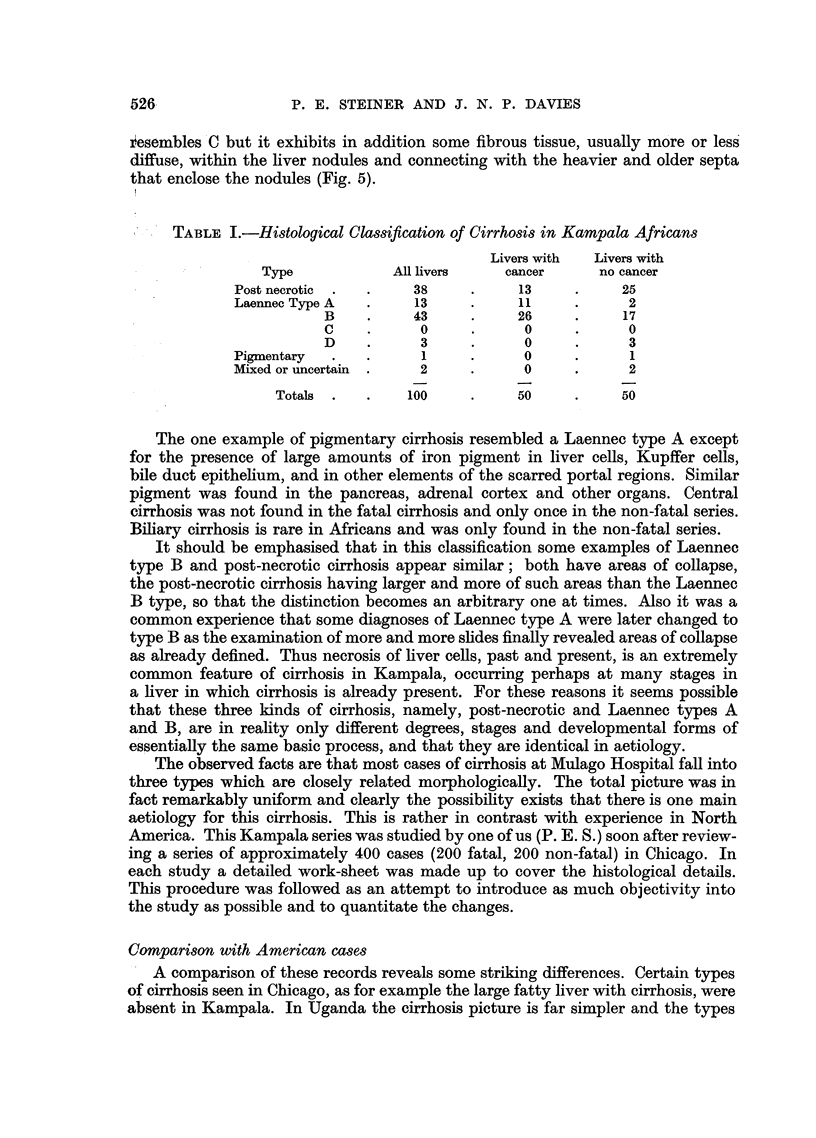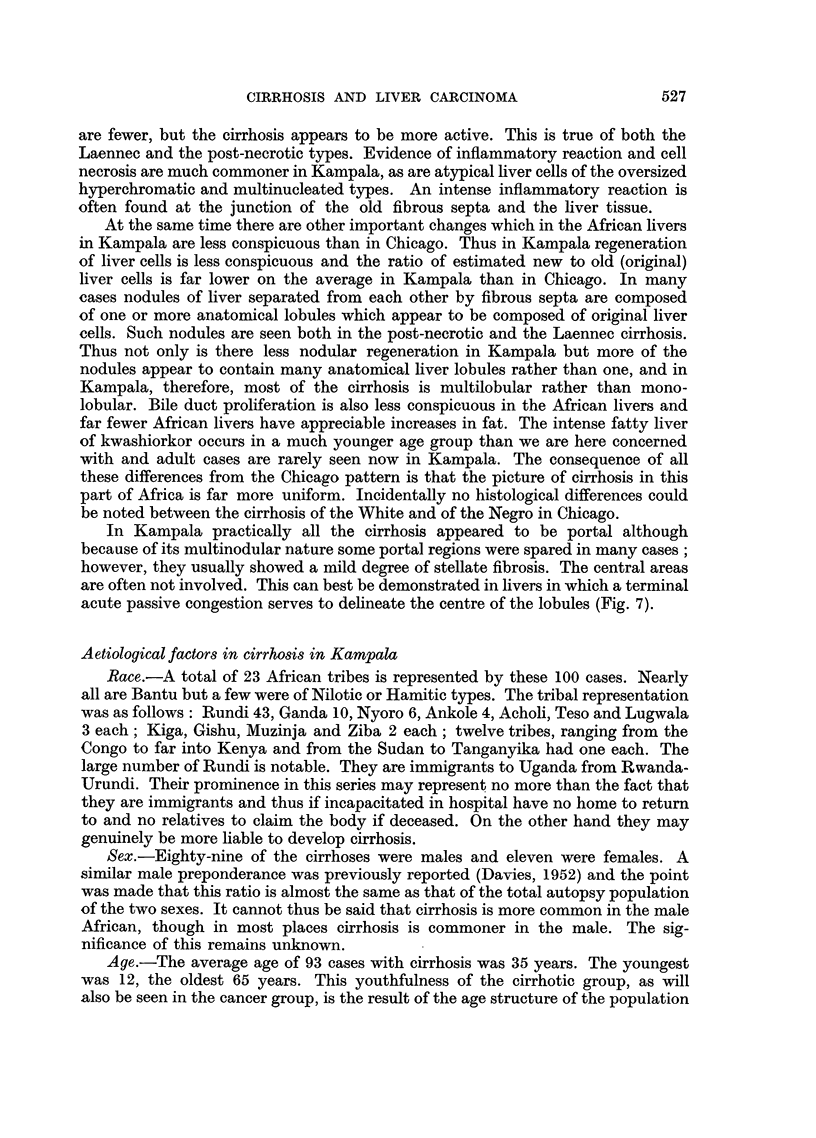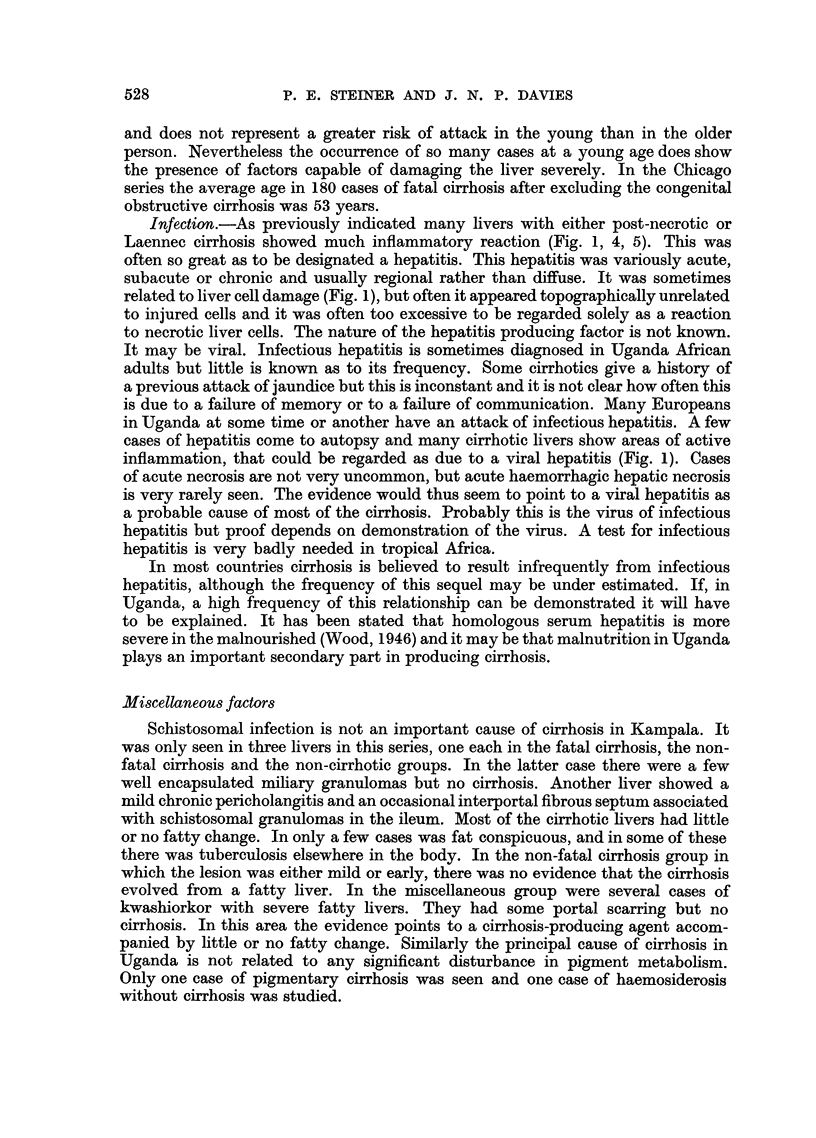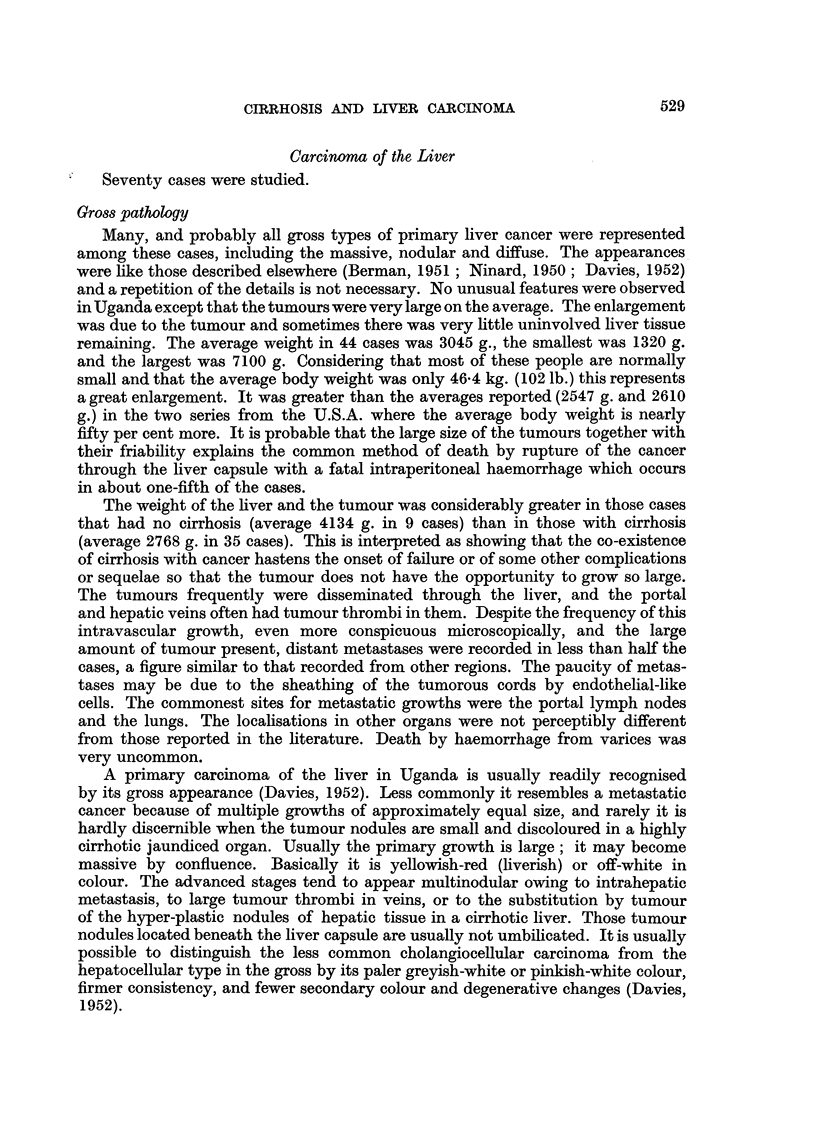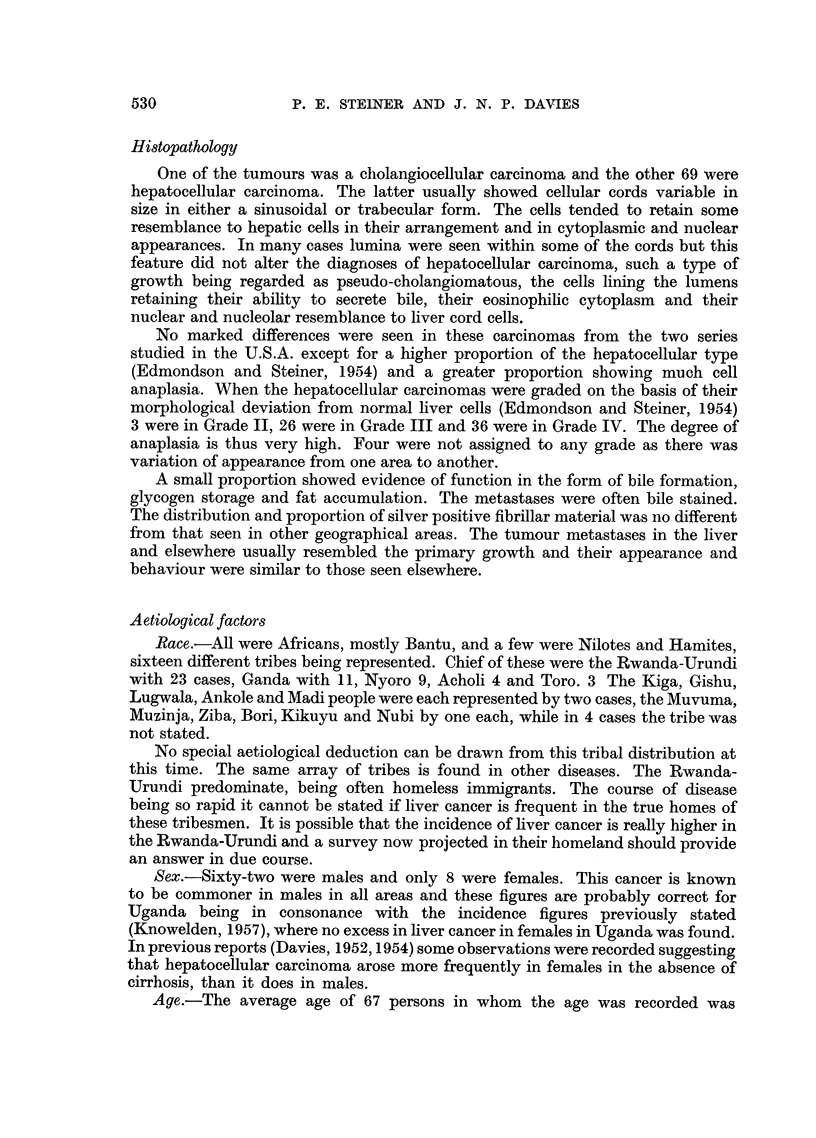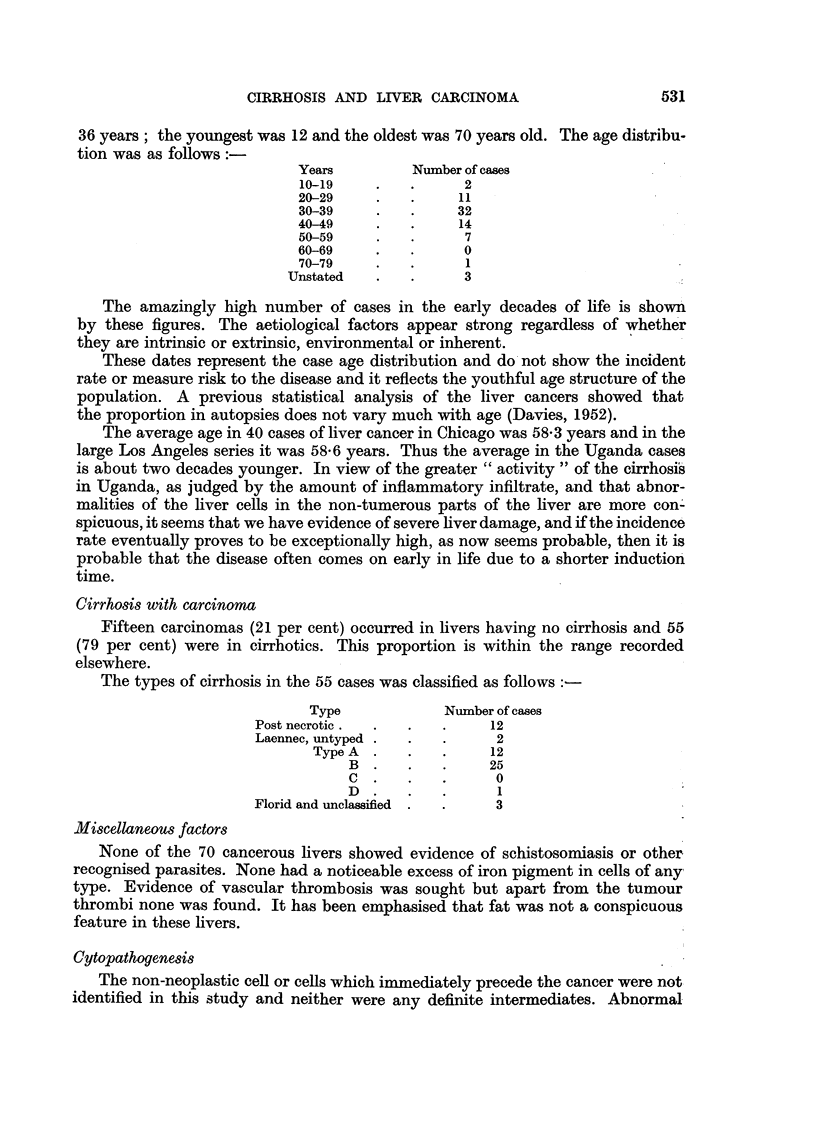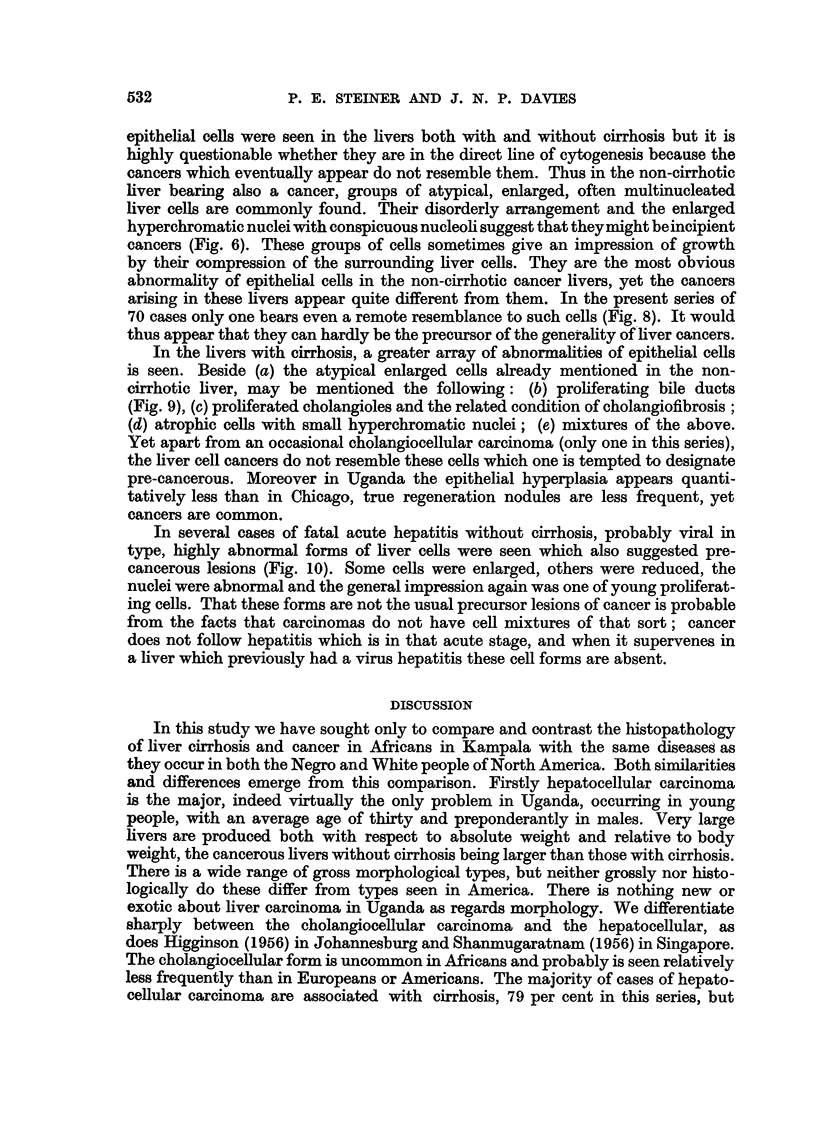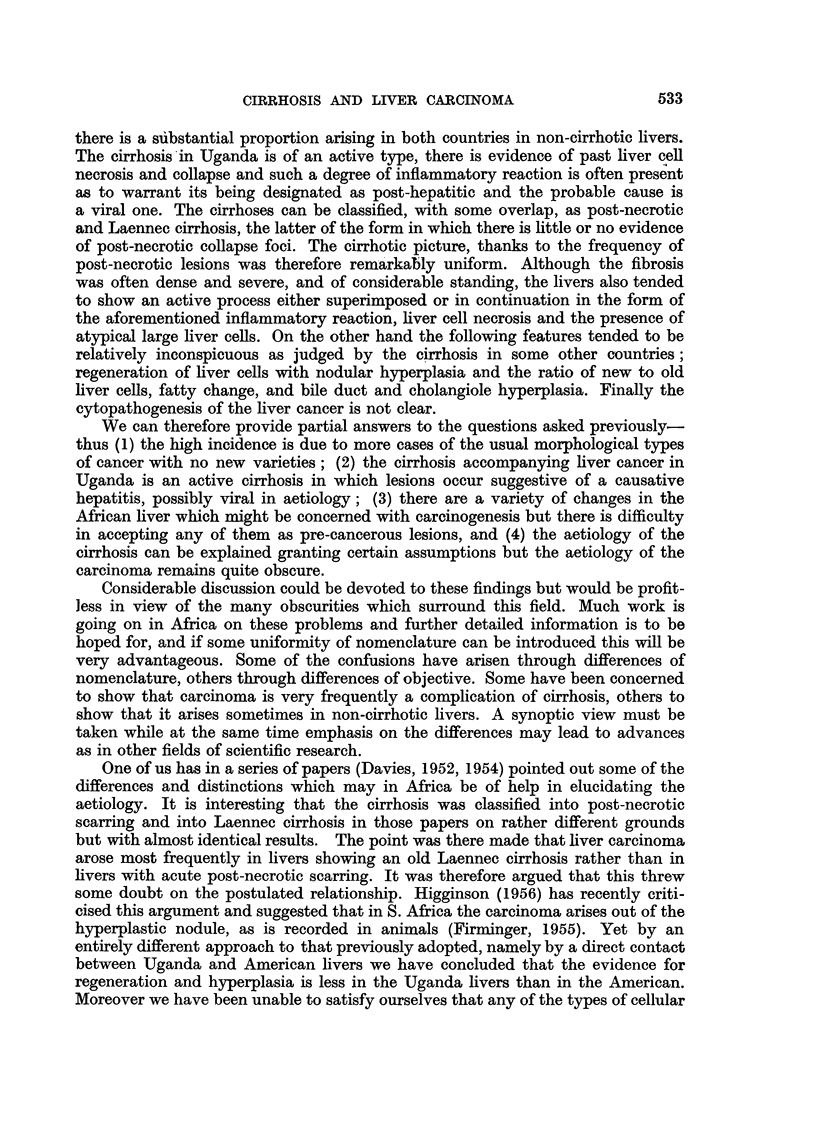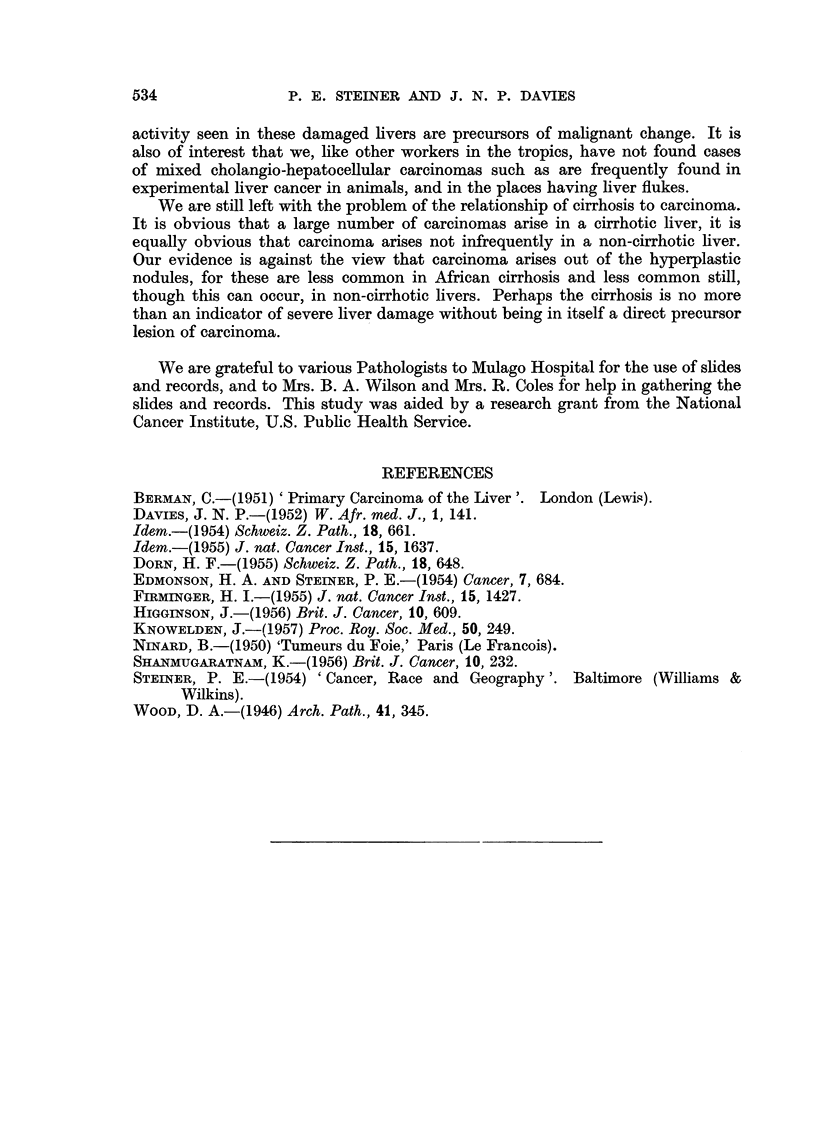# Cirrhosis and Primary Liver Carcinoma in Uganda Africans

**DOI:** 10.1038/bjc.1957.64

**Published:** 1957-12

**Authors:** P. E. Steiner, J. N. P. Davies

## Abstract

**Images:**


### CIRRHOSIS AND PRIMARY LIVER CARCINOMA IN UGANDA AFRICANS

P. E. STEINER
*From the Department of Pathology, University of Chicago*

AND

J. N. P. DAVIES
*From the Department of Pathology, Kampala Cancer Registry and Makerere College Medical School, Uganda*

Received for publication September 5, 1957

THERE is now mounting evidence to support the view that hepatocellular carcinoma occurs with undue frequency in the indigenous population of the African Continent. The relationship of cirrhosis to the development of carcinoma is not precisely established and studies to elucidate the nature of the relationship in Africa have only been undertaken in recent years. This report is based on a study of cases in the files of the Department of Pathology of the Makerere College Medical School on which autopsy examinations were performed at Mulago Hospital, Kampala during the years 1946–57. Not all the cases of cirrhosis or of carcinoma encountered in this period could be utilised, but the sample studied was representative and unbiased, being selected because of the ready availability of material and the mildness of the post-mortem changes. Because the sample is incomplete the statistical aspects are not included in the report. In any event they, as frequency studies, would soon be superseded by the results of an incidence rate study now being made. Preliminary results (Knowelden, 1957) suggest that the incidence of liver cancer in Uganda males is two and a half times that of a corresponding Danish population but that an elevated frequency in Uganda females could not be shown. Relative to other malignant tumours, liver cancers ranked fourth, being exceeded only by those of the uterine cervix, skin and penis (Davies, 1957). Carcinoma of the liver thus is an important and major neoplastic disease in Uganda. Most cancers arise in cirrhotic livers so that the cirrhosis also demands study.

This report will attempt to characterise the pathological features both of cirrhosis and of cancer in Uganda Africans. Because the ultimate objective is to find aetiological factors, special attention was paid during this study to the histopathogenesis of the lesions and to possible causative agents of both diseases. It is known from Africa (Berman, 1951) and other countries (Steiner, 1954) that where the frequency of liver cancers is high, the disease often comes on early in life. The higher yield of tumours and the possible shortened induction time suggest that the aetiological factors are intensified. A study of the exaggerated situation might reveal cause-and-effect relationships not apparent where the frequency is low.

Some special problems given consideration included the following :—

1. Is the high incidence of liver cancer due merely to more cases of the usual morphological types or do new varieties enter the picture ?

2. Is there anything peculiar or different about the cirrhosis, which is so often and so quickly associated with cancer ?

3. Is there anything discernible in the liver of the African which would explain the high frequencies of cancer and cirrhosis ? This thought arises from the observations of Dorn (1955) that the Negro and Caucasoid in the United States do not differ from each other appreciably either in the incidence of cirrhosis or of cancer.

4. Is the aetiology of either disease apparent or suggested by the morphological or the cytopathogenic picture ?

As a basis for contrast on some of these points, one of the authors (P. E. S.) has studied 137 cases of liver cancer in the U.S.A. [100 in Los Angeles (Edmondson and Steiner, 1954) and 37 in Chicago] and about 400 cases of cirrhosis occurring in some 10,000 autopsies on record in Chicago.

*Basis of the study*

This study is based on 70 cases of primary liver carcinoma and of 100 cases of advanced cirrhosis. In 50 of the cirrhotic livers a primary carcinoma was present. An additional 12 cases in which mild cirrhosis was an incidental post-mortem finding were also studied for the light they shed on histogenesis, but the main basis for the report of cirrhosis is the 100 advanced cases. About 100 other livers showing various other lesions, including hepatitis, kwashiorkor, acute necrosis, fatty change, schistosomiasis and other changes were studied and mention will be made of some of these cases later. All were native Africans *sensu stricto*, from a variety of tribes. All were autopsied cases ; the many liver cancers and cirrhosis cases diagnosed by biopsy were excluded. Classification was based on the microscopical sections ; in the majority of cases several slides were available and in many cases there were oversize sections, some up to lantern plate size, which made examination easy and accurate.

### *Cirrhosis in Kampala*

*Gross pathology*

The cirrhosis at Mulago Hospital has been previously described (Davies, 1952) and a repetition is here unnecessary except in so far as it sheds information on the the cancer problem and is essential to this report. The livers were usually small, firm, tawny and nodular. Fibrous adhesions bound the organ to adjacent structures

---

EXPLANATION OF PLATES.

FIG. 1.—Necroses and inflammatory cell change in post-necrotic cirrhosis : continuing activity. P.M. 170/54. × 50.

FIG. 2.—Laennec cirrhosis, Type A, to show connective tissue septa. P.M. 174/53. × 17.

FIG. 3.—Laennec cirrhosis Type A with central congestion. P.M. 354/56. Silver stain. × 50.

FIG. 4.—Laennec cirrhosis Type C. Showing large droplet fat in lesions of Laennec Type A cirrhosis. P.M. 25/54. × 50.

FIG. 5.—Laennec cirrhosis Type D, mild case. P.M. 159/52. × 50.

FIG. 6.—Liver cell atypia, in a case having a hepatocellular carcinoma. P.M. 299/55. × 210.

FIG. 7.—Laennec cirrhosis Type B with massive congestion. P.M. 10/53. × 24.

FIG. 8.—Hepacellular carcinoma Grade 4, having large cells. P.M. 91/51. × 290.

FIG. 9.—Cholangiofibrosis in a liver having both cancer and cirrhosis. P.M. 242/51. × 290.

FIG. 10.—Severe acute hepatitis, viral type. P.M. 152/53. × 65.

1

2

3

4

5

6

Steiner and Davies.

7

8

9

10

Steiner and Davies.

in many instances. The spleen was usually enlarged, often very greatly so, and firm, showing the microscopic features of chronic passive congestion. It was often adherent to the surrounding structures by massive adhesions in which were very large vascular channels. The portal system was enlarged and the collateral circulation was developed. Death was however usually not due to haemorrhage from varices, perhaps because the dense adhesions provide safety valves, but to liver failure and coma or to intercurrent infection.

The liver was usually small; never was it greatly enlarged. The average weight in 40 cases without cancer was only 1237 g., the minimum was 565 g. and the maximum was 2675 g. The body weight at autopsy averaged 49·1 kg. (108 lb.). The nodularity was variable in size but was usually coarse; 15 mm. was a commonly recorded measurement for the larger nodules. The amount of scarring and its distribution likewise was variable but was usually severe. On the cut surface it was recorded a number of times as comprising about half the area. Grossly many livers were described as typical hobnail and diagnosed as Laennec cirrhosis. In others the scarring and nodularity were more severe, or affected one lobe more than the other, and the diagnosis was that of post-necrotic scarring. In the more typical cases of each the diagnosis was easy. Often however, the gross pictures merged and the diagnosis was a " toss-up ".

The liver weight averaged the same in the post-necrotic cirrhosis (1248 g.) as in the Laennec type B (1253 g.) to be described later. The other groups were not large enough to warrant comparisons but the livers seemed to be within the same range, except in the Laennec type A class where they averaged rather heavier.

*Histopathology*

A histological classification of the types of cirrhosis is given in Table I. It is clear that the great majority of cases are of either the post-necrotic type or the Laennec type, the latter predominating. Few other types were represented; one was a pigmentary cirrhosis (haemochromatosis) and two were of uncertain or mixed types.

The basis for this classification will be elaborated elsewhere. For purposes of this report it is sufficient to state that post-necrotic cirrhosis is that form which shows fibrous scars and bands, which are usually broad and which show evidence of collapse and condensation of liver stroma in the form of groups of blood vessels and bile ducts within the larger scars. The surviving liver tissue shows greater or lesser degrees of regenerative hyperplasia. The scarring and regeneration result in a distortion of the liver architecture. There is almost always evidence of the continuing activity of the process to be found at one place or another in the form of liver cell necrosis and acute to chronic inflammatory reaction involving either original liver tissue or regenerated nodules (Fig. 1).

The Laennec type A is the simplest chronic cirrhosis. It has bands or septa of connective tissue which subdivide the organ into nodules of various sizes which show also various amounts of regenerative hyperplasia, the whole resulting in a distorted liver architecture (Fig. 2, 3). The Laennec type B resembles type A but in addition shows small groups of blood vessels and bile ducts here and there within the fibrous septa as evidence of probable previous collapse of liver lobules and condensation of the stroma residues. Laennec type C resembles A or B but it has in addition much large-droplet fat in liver cells (Fig. 4). Laennec type D

resembles C but it exhibits in addition some fibrous tissue, usually more or less diffuse, within the liver nodules and connecting with the heavier and older septa that enclose the nodules (Fig. 5).

TABLE I.—*Histological Classification of Cirrhosis in Kampala Africans*

| Type | All livers | Livers with cancer | Livers with no cancer |
|---|---|---|---|
| Post necrotic | 38 | 13 | 25 |
| Laennec Type A | 13 | 11 | 2 |
| B | 43 | 26 | 17 |
| C | 0 | 0 | 0 |
| D | 3 | 0 | 3 |
| Pigmentary | 1 | 0 | 1 |
| Mixed or uncertain | 2 | 0 | 2 |
| Totals | 100 | 50 | 50 |

The one example of pigmentary cirrhosis resembled a Laennec type A except for the presence of large amounts of iron pigment in liver cells, Kupffer cells, bile duct epithelium, and in other elements of the scarred portal regions. Similar pigment was found in the pancreas, adrenal cortex and other organs. Central cirrhosis was not found in the fatal cirrhosis and only once in the non-fatal series. Biliary cirrhosis is rare in Africans and was only found in the non-fatal series.

It should be emphasised that in this classification some examples of Laennec type B and post-necrotic cirrhosis appear similar ; both have areas of collapse, the post-necrotic cirrhosis having larger and more of such areas than the Laennec B type, so that the distinction becomes an arbitrary one at times. Also it was a common experience that some diagnoses of Laennec type A were later changed to type B as the examination of more and more slides finally revealed areas of collapse as already defined. Thus necrosis of liver cells, past and present, is an extremely common feature of cirrhosis in Kampala, occurring perhaps at many stages in a liver in which cirrhosis is already present. For these reasons it seems possible that these three kinds of cirrhosis, namely, post-necrotic and Laennec types A and B, are in reality only different degrees, stages and developmental forms of essentially the same basic process, and that they are identical in aetiology.

The observed facts are that most cases of cirrhosis at Mulago Hospital fall into three types which are closely related morphologically. The total picture was in fact remarkably uniform and clearly the possibility exists that there is one main aetiology for this cirrhosis. This is rather in contrast with experience in North America. This Kampala series was studied by one of us (P. E. S.) soon after reviewing a series of approximately 400 cases (200 fatal, 200 non-fatal) in Chicago. In each study a detailed work-sheet was made up to cover the histological details. This procedure was followed as an attempt to introduce as much objectivity into the study as possible and to quantitate the changes.

*Comparison with American cases*

A comparison of these records reveals some striking differences. Certain types of cirrhosis seen in Chicago, as for example the large fatty liver with cirrhosis, were absent in Kampala. In Uganda the cirrhosis picture is far simpler and the types are fewer, but the cirrhosis appears to be more active. This is true of both the Laennec and the post-necrotic types. Evidence of inflammatory reaction and cell necrosis are much commoner in Kampala, as are atypical liver cells of the oversized hyperchromatic and multinucleated types. An intense inflammatory reaction is often found at the junction of the old fibrous septa and the liver tissue.

At the same time there are other important changes which in the African livers in Kampala are less conspicuous than in Chicago. Thus in Kampala regeneration of liver cells is less conspicuous and the ratio of estimated new to old (original) liver cells is far lower on the average in Kampala than in Chicago. In many cases nodules of liver separated from each other by fibrous septa are composed of one or more anatomical lobules which appear to be composed of original liver cells. Such nodules are seen both in the post-necrotic and the Laennec cirrhosis. Thus not only is there less nodular regeneration in Kampala but more of the nodules appear to contain many anatomical liver lobules rather than one, and in Kampala, therefore, most of the cirrhosis is multilobular rather than monolobular. Bile duct proliferation is also less conspicuous in the African livers and far fewer African livers have appreciable increases in fat. The intense fatty liver of kwashiorkor occurs in a much younger age group than we are here concerned with and adult cases are rarely seen now in Kampala. The consequence of all these differences from the Chicago pattern is that the picture of cirrhosis in this part of Africa is far more uniform. Incidentally no histological differences could be noted between the cirrhosis of the White and of the Negro in Chicago.

In Kampala practically all the cirrhosis appeared to be portal although because of its multinodular nature some portal regions were spared in many cases ; however, they usually showed a mild degree of stellate fibrosis. The central areas are often not involved. This can best be demonstrated in livers in which a terminal acute passive congestion serves to delineate the centre of the lobules (Fig. 7).

*Aetiological factors in cirrhosis in Kampala*

*Race.*—A total of 23 African tribes is represented by these 100 cases. Nearly all are Bantu but a few were of Nilotic or Hamitic types. The tribal representation was as follows : Rundi 43, Ganda 10, Nyoro 6, Ankole 4, Acholi, Teso and Lugwala 3 each ; Kiga, Gishu, Muzinja and Ziba 2 each ; twelve tribes, ranging from the Congo to far into Kenya and from the Sudan to Tanganyika had one each. The large number of Rundi is notable. They are immigrants to Uganda from Rwanda-Urundi. Their prominence in this series may represent no more than the fact that they are immigrants and thus if incapacitated in hospital have no home to return to and no relatives to claim the body if deceased. On the other hand they may genuinely be more liable to develop cirrhosis.

*Sex.*—Eighty-nine of the cirrhoses were males and eleven were females. A similar male preponderance was previously reported (Davies, 1952) and the point was made that this ratio is almost the same as that of the total autopsy population of the two sexes. It cannot thus be said that cirrhosis is more common in the male African, though in most places cirrhosis is commoner in the male. The significance of this remains unknown.

*Age.*—The average age of 93 cases with cirrhosis was 35 years. The youngest was 12, the oldest 65 years. This youthfulness of the cirrhotic group, as will also be seen in the cancer group, is the result of the age structure of the population and does not represent a greater risk of attack in the young than in the older person. Nevertheless the occurrence of so many cases at a young age does show the presence of factors capable of damaging the liver severely. In the Chicago series the average age in 180 cases of fatal cirrhosis after excluding the congenital obstructive cirrhosis was 53 years.

*Infection.*—As previously indicated many livers with either post-necrotic or Laennec cirrhosis showed much inflammatory reaction (Fig. 1, 4, 5). This was often so great as to be designated a hepatitis. This hepatitis was variously acute, subacute or chronic and usually regional rather than diffuse. It was sometimes related to liver cell damage (Fig. 1), but often it appeared topographically unrelated to injured cells and it was often too excessive to be regarded solely as a reaction to necrotic liver cells. The nature of the hepatitis producing factor is not known. It may be viral. Infectious hepatitis is sometimes diagnosed in Uganda African adults but little is known as to its frequency. Some cirrhotics give a history of a previous attack of jaundice but this is inconstant and it is not clear how often this is due to a failure of memory or to a failure of communication. Many Europeans in Uganda at some time or another have an attack of infectious hepatitis. A few cases of hepatitis come to autopsy and many cirrhotic livers show areas of active inflammation, that could be regarded as due to a viral hepatitis (Fig. 1). Cases of acute necrosis are not very uncommon, but acute haemorrhagic hepatic necrosis is very rarely seen. The evidence would thus seem to point to a viral hepatitis as a probable cause of most of the cirrhosis. Probably this is the virus of infectious hepatitis but proof depends on demonstration of the virus. A test for infectious hepatitis is very badly needed in tropical Africa.

In most countries cirrhosis is believed to result infrequently from infectious hepatitis, although the frequency of this sequel may be under estimated. If, in Uganda, a high frequency of this relationship can be demonstrated it will have to be explained. It has been stated that homologous serum hepatitis is more severe in the malnourished (Wood, 1946) and it may be that malnutrition in Uganda plays an important secondary part in producing cirrhosis.

### *Miscellaneous factors*

Schistosomal infection is not an important cause of cirrhosis in Kampala. It was only seen in three livers in this series, one each in the fatal cirrhosis, the non-fatal cirrhosis and the non-cirrhotic groups. In the latter case there were a few well encapsulated miliary granulomas but no cirrhosis. Another liver showed a mild chronic pericholangitis and an occasional interportal fibrous septum associated with schistosomal granulomas in the ileum. Most of the cirrhotic livers had little or no fatty change. In only a few cases was fat conspicuous, and in some of these there was tuberculosis elsewhere in the body. In the non-fatal cirrhosis group in which the lesion was either mild or early, there was no evidence that the cirrhosis evolved from a fatty liver. In the miscellaneous group were several cases of kwashiorkor with severe fatty livers. They had some portal scarring but no cirrhosis. In this area the evidence points to a cirrhosis-producing agent accompanied by little or no fatty change. Similarly the principal cause of cirrhosis in Uganda is not related to any significant disturbance in pigment metabolism. Only one case of pigmentary cirrhosis was seen and one case of haemosiderosis without cirrhosis was studied.

### *Carcinoma of the Liver*

Seventy cases were studied.

### *Gross pathology*

Many, and probably all gross types of primary liver cancer were represented among these cases, including the massive, nodular and diffuse. The appearances were like those described elsewhere (Berman, 1951 ; Ninard, 1950 ; Davies, 1952) and a repetition of the details is not necessary. No unusual features were observed in Uganda except that the tumours were very large on the average. The enlargement was due to the tumour and sometimes there was very little uninvolved liver tissue remaining. The average weight in 44 cases was 3045 g., the smallest was 1320 g. and the largest was 7100 g. Considering that most of these people are normally small and that the average body weight was only 46·4 kg. (102 lb.) this represents a great enlargement. It was greater than the averages reported (2547 g. and 2610 g.) in the two series from the U.S.A. where the average body weight is nearly fifty per cent more. It is probable that the large size of the tumours together with their friability explains the common method of death by rupture of the cancer through the liver capsule with a fatal intraperitoneal haemorrhage which occurs in about one-fifth of the cases.

The weight of the liver and the tumour was considerably greater in those cases that had no cirrhosis (average 4134 g. in 9 cases) than in those with cirrhosis (average 2768 g. in 35 cases). This is interpreted as showing that the co-existence of cirrhosis with cancer hastens the onset of failure or of some other complications or sequelae so that the tumour does not have the opportunity to grow so large. The tumours frequently were disseminated through the liver, and the portal and hepatic veins often had tumour thrombi in them. Despite the frequency of this intravascular growth, even more conspicuous microscopically, and the large amount of tumour present, distant metastases were recorded in less than half the cases, a figure similar to that recorded from other regions. The paucity of metastases may be due to the sheathing of the tumorous cords by endothelial-like cells. The commonest sites for metastatic growths were the portal lymph nodes and the lungs. The localisations in other organs were not perceptibly different from those reported in the literature. Death by haemorrhage from varices was very uncommon.

A primary carcinoma of the liver in Uganda is usually readily recognised by its gross appearance (Davies, 1952). Less commonly it resembles a metastatic cancer because of multiple growths of approximately equal size, and rarely it is hardly discernible when the tumour nodules are small and discoloured in a highly cirrhotic jaundiced organ. Usually the primary growth is large ; it may become massive by confluence. Basically it is yellowish-red (liverish) or off-white in colour. The advanced stages tend to appear multinodular owing to intrahepatic metastasis, to large tumour thrombi in veins, or to the substitution by tumour of the hyper-plastic nodules of hepatic tissue in a cirrhotic liver. Those tumour nodules located beneath the liver capsule are usually not umbilicated. It is usually possible to distinguish the less common cholangiocellular carcinoma from the hepatocellular type in the gross by its paler greyish-white or pinkish-white colour, firmer consistency, and fewer secondary colour and degenerative changes (Davies, 1952).

*Histopathology*

One of the tumours was a cholangiocellular carcinoma and the other 69 were hepatocellular carcinoma. The latter usually showed cellular cords variable in size in either a sinusoidal or trabecular form. The cells tended to retain some resemblance to hepatic cells in their arrangement and in cytoplasmic and nuclear appearances. In many cases lumina were seen within some of the cords but this feature did not alter the diagnoses of hepatocellular carcinoma, such a type of growth being regarded as pseudo-cholangiomatous, the cells lining the lumens retaining their ability to secrete bile, their eosinophilic cytoplasm and their nuclear and nucleolar resemblance to liver cord cells.

No marked differences were seen in these carcinomas from the two series studied in the U.S.A. except for a higher proportion of the hepatocellular type (Edmondson and Steiner, 1954) and a greater proportion showing much cell anaplasia. When the hepatocellular carcinomas were graded on the basis of their morphological deviation from normal liver cells (Edmondson and Steiner, 1954) 3 were in Grade II, 26 were in Grade III and 36 were in Grade IV. The degree of anaplasia is thus very high. Four were not assigned to any grade as there was variation of appearance from one area to another.

A small proportion showed evidence of function in the form of bile formation, glycogen storage and fat accumulation. The metastases were often bile stained. The distribution and proportion of silver positive fibrillar material was no different from that seen in other geographical areas. The tumour metastases in the liver and elsewhere usually resembled the primary growth and their appearance and behaviour were similar to those seen elsewhere.

*Aetiological factors*

*Race.*—All were Africans, mostly Bantu, and a few were Nilotes and Hamites, sixteen different tribes being represented. Chief of these were the Rwanda-Urundi with 23 cases, Ganda with 11, Nyoro 9, Acholi 4 and Toro. 3 The Kiga, Gishu, Lugwala, Ankole and Madi people were each represented by two cases, the Muvuma, Muzinja, Ziba, Bori, Kikuyu and Nubi by one each, while in 4 cases the tribe was not stated.

No special aetiological deduction can be drawn from this tribal distribution at this time. The same array of tribes is found in other diseases. The Rwanda-Urundi predominate, being often homeless immigrants. The course of disease being so rapid it cannot be stated if liver cancer is frequent in the true homes of these tribesmen. It is possible that the incidence of liver cancer is really higher in the Rwanda-Urundi and a survey now projected in their homeland should provide an answer in due course.

*Sex.*—Sixty-two were males and only 8 were females. This cancer is known to be commoner in males in all areas and these figures are probably correct for Uganda being in consonance with the incidence figures previously stated (Knowelden, 1957), where no excess in liver cancer in females in Uganda was found. In previous reports (Davies, 1952, 1954) some observations were recorded suggesting that hepatocellular carcinoma arose more frequently in females in the absence of cirrhosis, than it does in males.

*Age.*—The average age of 67 persons in whom the age was recorded was

36 years ; the youngest was 12 and the oldest was 70 years old. The age distribution was as follows :—

| Years | Number of cases |
|---|---|
| 10–19 | 2 |
| 20–29 | 11 |
| 30–39 | 32 |
| 40–49 | 14 |
| 50–59 | 7 |
| 60–69 | 0 |
| 70–79 | 1 |
| Unstated | 3 |

The amazingly high number of cases in the early decades of life is shown by these figures. The aetiological factors appear strong regardless of whether they are intrinsic or extrinsic, environmental or inherent.

These dates represent the case age distribution and do not show the incident rate or measure risk to the disease and it reflects the youthful age structure of the population. A previous statistical analysis of the liver cancers showed that the proportion in autopsies does not vary much with age (Davies, 1952).

The average age in 40 cases of liver cancer in Chicago was 58·3 years and in the large Los Angeles series it was 58·6 years. Thus the average in the Uganda cases is about two decades younger. In view of the greater " activity " of the cirrhosis in Uganda, as judged by the amount of inflammatory infiltrate, and that abnormalities of the liver cells in the non-tumerous parts of the liver are more conspicuous, it seems that we have evidence of severe liver damage, and if the incidence rate eventually proves to be exceptionally high, as now seems probable, then it is probable that the disease often comes on early in life due to a shorter induction time.

*Cirrhosis with carcinoma*

Fifteen carcinomas (21 per cent) occurred in livers having no cirrhosis and 55 (79 per cent) were in cirrhotics. This proportion is within the range recorded elsewhere.

The types of cirrhosis in the 55 cases was classified as follows :—

| Type | Number of cases |
|---|---|
| Post necrotic | 12 |
| Laennec, untyped | 2 |
| Type A | 12 |
| B | 25 |
| C | 0 |
| D | 1 |
| Florid and unclassified | 3 |

*Miscellaneous factors*

None of the 70 cancerous livers showed evidence of schistosomiasis or other recognised parasites. None had a noticeable excess of iron pigment in cells of any type. Evidence of vascular thrombosis was sought but apart from the tumour thrombi none was found. It has been emphasised that fat was not a conspicuous feature in these livers.

*Cytopathogenesis*

The non-neoplastic cell or cells which immediately precede the cancer were not identified in this study and neither were any definite intermediates. Abnormal epithelial cells were seen in the livers both with and without cirrhosis but it is highly questionable whether they are in the direct line of cytogenesis because the cancers which eventually appear do not resemble them. Thus in the non-cirrhotic liver bearing also a cancer, groups of atypical, enlarged, often multinucleated liver cells are commonly found. Their disorderly arrangement and the enlarged hyperchromatic nuclei with conspicuous nucleoli suggest that they might be incipient cancers (Fig. 6). These groups of cells sometimes give an impression of growth by their compression of the surrounding liver cells. They are the most obvious abnormality of epithelial cells in the non-cirrhotic cancer livers, yet the cancers arising in these livers appear quite different from them. In the present series of 70 cases only one bears even a remote resemblance to such cells (Fig. 8). It would thus appear that they can hardly be the precursor of the generality of liver cancers.

In the livers with cirrhosis, a greater array of abnormalities of epithelial cells is seen. Beside (*a*) the atypical enlarged cells already mentioned in the non-cirrhotic liver, may be mentioned the following: (*b*) proliferating bile ducts (Fig. 9), (*c*) proliferated cholangioles and the related condition of cholangiofibrosis; (*d*) atrophic cells with small hyperchromatic nuclei; (*e*) mixtures of the above. Yet apart from an occasional cholangiocellular carcinoma (only one in this series), the liver cell cancers do not resemble these cells which one is tempted to designate pre-cancerous. Moreover in Uganda the epithelial hyperplasia appears quantitatively less than in Chicago, true regeneration nodules are less frequent, yet cancers are common.

In several cases of fatal acute hepatitis without cirrhosis, probably viral in type, highly abnormal forms of liver cells were seen which also suggested pre-cancerous lesions (Fig. 10). Some cells were enlarged, others were reduced, the nuclei were abnormal and the general impression again was one of young proliferating cells. That these forms are not the usual precursor lesions of cancer is probable from the facts that carcinomas do not have cell mixtures of that sort; cancer does not follow hepatitis which is in that acute stage, and when it supervenes in a liver which previously had a virus hepatitis these cell forms are absent.

### DISCUSSION

In this study we have sought only to compare and contrast the histopathology of liver cirrhosis and cancer in Africans in Kampala with the same diseases as they occur in both the Negro and White people of North America. Both similarities and differences emerge from this comparison. Firstly hepatocellular carcinoma is the major, indeed virtually the only problem in Uganda, occurring in young people, with an average age of thirty and preponderantly in males. Very large livers are produced both with respect to absolute weight and relative to body weight, the cancerous livers without cirrhosis being larger than those with cirrhosis. There is a wide range of gross morphological types, but neither grossly nor histologically do these differ from types seen in America. There is nothing new or exotic about liver carcinoma in Uganda as regards morphology. We differentiate sharply between the cholangiocellular carcinoma and the hepatocellular, as does Higginson (1956) in Johannesburg and Shanmugaratnam (1956) in Singapore. The cholangiocellular form is uncommon in Africans and probably is seen relatively less frequently than in Europeans or Americans. The majority of cases of hepatocellular carcinoma are associated with cirrhosis, 79 per cent in this series, but there is a substantial proportion arising in both countries in non-cirrhotic livers. The cirrhosis in Uganda is of an active type, there is evidence of past liver cell necrosis and collapse and such a degree of inflammatory reaction is often present as to warrant its being designated as post-hepatitic and the probable cause is a viral one. The cirrhoses can be classified, with some overlap, as post-necrotic and Laennec cirrhosis, the latter of the form in which there is little or no evidence of post-necrotic collapse foci. The cirrhotic picture, thanks to the frequency of post-necrotic lesions was therefore remarkably uniform. Although the fibrosis was often dense and severe, and of considerable standing, the livers also tended to show an active process either superimposed or in continuation in the form of the aforementioned inflammatory reaction, liver cell necrosis and the presence of atypical large liver cells. On the other hand the following features tended to be relatively inconspicuous as judged by the cirrhosis in some other countries; regeneration of liver cells with nodular hyperplasia and the ratio of new to old liver cells, fatty change, and bile duct and cholangiole hyperplasia. Finally the cytopathogenesis of the liver cancer is not clear.

We can therefore provide partial answers to the questions asked previously—thus (1) the high incidence is due to more cases of the usual morphological types of cancer with no new varieties; (2) the cirrhosis accompanying liver cancer in Uganda is an active cirrhosis in which lesions occur suggestive of a causative hepatitis, possibly viral in aetiology; (3) there are a variety of changes in the African liver which might be concerned with carcinogenesis but there is difficulty in accepting any of them as pre-cancerous lesions, and (4) the aetiology of the cirrhosis can be explained granting certain assumptions but the aetiology of the carcinoma remains quite obscure.

Considerable discussion could be devoted to these findings but would be profitless in view of the many obscurities which surround this field. Much work is going on in Africa on these problems and further detailed information is to be hoped for, and if some uniformity of nomenclature can be introduced this will be very advantageous. Some of the confusions have arisen through differences of nomenclature, others through differences of objective. Some have been concerned to show that carcinoma is very frequently a complication of cirrhosis, others to show that it arises sometimes in non-cirrhotic livers. A synoptic view must be taken while at the same time emphasis on the differences may lead to advances as in other fields of scientific research.

One of us has in a series of papers (Davies, 1952, 1954) pointed out some of the differences and distinctions which may in Africa be of help in elucidating the aetiology. It is interesting that the cirrhosis was classified into post-necrotic scarring and into Laennec cirrhosis in those papers on rather different grounds but with almost identical results. The point was there made that liver carcinoma arose most frequently in livers showing an old Laennec cirrhosis rather than in livers with acute post-necrotic scarring. It was therefore argued that this threw some doubt on the postulated relationship. Higginson (1956) has recently criticised this argument and suggested that in S. Africa the carcinoma arises out of the hyperplastic nodule, as is recorded in animals (Firminger, 1955). Yet by an entirely different approach to that previously adopted, namely by a direct contact between Uganda and American livers we have concluded that the evidence for regeneration and hyperplasia is less in the Uganda livers than in the American. Moreover we have been unable to satisfy ourselves that any of the types of cellular activity seen in these damaged livers are precursors of malignant change. It is also of interest that we, like other workers in the tropics, have not found cases of mixed cholangio-hepatocellular carcinomas such as are frequently found in experimental liver cancer in animals, and in the places having liver flukes.

We are still left with the problem of the relationship of cirrhosis to carcinoma. It is obvious that a large number of carcinomas arise in a cirrhotic liver, it is equally obvious that carcinoma arises not infrequently in a non-cirrhotic liver. Our evidence is against the view that carcinoma arises out of the hyperplastic nodules, for these are less common in African cirrhosis and less common still, though this can occur, in non-cirrhotic livers. Perhaps the cirrhosis is no more than an indicator of severe liver damage without being in itself a direct precursor lesion of carcinoma.

We are grateful to various Pathologists to Mulago Hospital for the use of slides and records, and to Mrs. B. A. Wilson and Mrs. R. Coles for help in gathering the slides and records. This study was aided by a research grant from the National Cancer Institute, U.S. Public Health Service.

### REFERENCES

BERMAN, C.—(1951) ' Primary Carcinoma of the Liver '. London (Lewis).
DAVIES, J. N. P.—(1952) *W. Afr. med. J.*, **1**, 141.
*Idem.*—(1954) *Schweiz. Z. Path.*, **18**, 661.
*Idem.*—(1955) *J. nat. Cancer Inst.*, **15**, 1637.
DORN, H. F.—(1955) *Schweiz. Z. Path.*, **18**, 648.
EDMONSON, H. A. AND STEINER, P. E.—(1954) *Cancer*, **7**, 684.
FIRMINGER, H. I.—(1955) *J. nat. Cancer Inst.*, **15**, 1427.
HIGGINSON, J.—(1956) *Brit. J. Cancer*, **10**, 609.
KNOWELDEN, J.—(1957) *Proc. Roy. Soc. Med.*, **50**, 249.
NINARD, B.—(1950) 'Tumeurs du Foie,' Paris (Le Francois).
SHANMUGARATNAM, K.—(1956) *Brit. J. Cancer*, **10**, 232.
STEINER, P. E.—(1954) ' Cancer, Race and Geography '. Baltimore (Williams & Wilkins).
WOOD, D. A.—(1946) *Arch. Path.*, **41**, 345.